# Clinician's Attitudes to the Introduction of Routine Weighing in Pregnancy

**DOI:** 10.1155/2016/2049673

**Published:** 2016-06-30

**Authors:** Tim Hasted, Helen Stapleton, Michael M. Beckmann, Shelley A. Wilkinson

**Affiliations:** ^1^Department of Obstetrics and Gynaecology, Mater Health Services, Brisbane, QLD 4101, Australia; ^2^University of Queensland School of Medicine, Brisbane, QLD 4006, Australia; ^3^Midwifery Research Unit, University of Queensland School of Nursing and Midwifery, Brisbane, QLD 4072, Australia; ^4^Mater Research Institute, University of Queensland, Brisbane, QLD 4101, Australia; ^5^Department of Nutrition and Dietetics, Mater Health Services, Brisbane, QLD 4101, Australia

## Abstract

*Background.* Excessive gestational weight gain poses significant short- and long-term health risks to both mother and baby. Professional bodies and health services increasingly recommend greater attention be paid to weight gain in pregnancy. A large Australian tertiary maternity hospital plans to facilitate the (re)introduction of routine weighing of all women at every antenatal visit.* Objective.* To identify clinicians' perspectives of barriers and enablers to routinely weighing pregnant women and variations in current practice, knowledge, and attitudes between different staff groups.* Method.* Forty-four maternity staff from three professional groups were interviewed in four focus groups. Staff included midwives; medical staff; and dietitians. Transcripts underwent qualitative content analysis to identify and examine barriers and enablers to the routine weighing of women throughout pregnancy.* Results.* While most staff supported routine weighing, various concerns were raised. Issues included access to resources and staff; the ability to provide appropriate counselling and evidence-based interventions; and the impact of weighing on patients and the therapeutic relationship.* Conclusion*. Many clinicians supported the practice of routine weighing in pregnancy, but barriers were also identified. Implementation strategies will be tailored to the discrete professional groups and will address identified gaps in knowledge, resources, and clinician skills and confidence.

## 1. Introduction

Excessive gestational weight gain (eGWG) for women of any prepregnancy Body Mass Index (BMI) is associated with adverse maternal and infant outcomes, including diabetes, preeclampsia, caesarean section, fetal macrosomia, admission to neonatal nursery, increased risk of postpartum weight retention, and risk of chronic disease for both mother and baby [1–6].

Routine weighing of women throughout their pregnancy used to be standard practice. In the first half of the twentieth century weight monitoring was conducted primarily to ensure that patients complied with the then common policy of encouraging weight restriction with target weights considerably lower than today. It was thought that excess weight gain caused preeclampsia, complicated births, and obesity [7]. In the 1960s and 1970s it became clear that this focus on weight restriction contributed to increased rates of low birth weight infants and associated morbidity and mortality. Target weights were adjusted and regular weighing continued with the new rationale that it was to ensure that women put on enough weight [8]. In the 1990s the practice was challenged after studies found that monitoring weight was not particularly effective in identifying women who would give birth to infants small for gestational age or for the development of preeclampsia. Clinicians argued that “routine weighing of patients may produce unnecessary anxiety and should cease” [9, 10]. In many jurisdictions routine weighing was replaced by a once-off calculation of a woman's BMI at the initial visit only, with the prevailing view that “maternal weight change is not a clinically useful screening tool for detection of growth restriction, macrosomia or pre-eclampsia” [11].

Informed by a growing evidence base, international guidelines are increasingly suggesting that assessment and promotion of appropriate gestational weight gain (GWG) should be reintroduced as a part of routine antenatal care for all women [12–15]. The revised US Institute of Medicine's (IOM) guidelines state that “health care providers should chart women's weight gain and share the results with them so that they become aware of their progress” [12]. Canadian guidelines suggest that health professionals “can use weight monitoring tools to assess the progress of pregnancy, track a woman's weight gain over time and identify unusual patterns of weight gain earlier in pregnancy… A single measure is not enough to determine whether weight gain is on track” [14]. Australian guidelines state that “assessment of appropriate weight gain should form part of routine care for all women” [15].

Routine weighing may help facilitate conversations about weight, enable more appropriate goal setting, and lead to more accurate measurement, and it may reduce GWG for all women. It has been shown that if health practitioners do not raise the issue of weight, women perceive it as not important [16]. The evidence indicates that it also matters who weighs women, with self-reporting being inaccurate. Furthermore, overweight and obese women are much more likely to overestimate appropriate weight gain compared to healthy weight women [17] and to underreport their weight [18]. Recommended target weight gains for pregnant women have been strongly associated with actual weight gain [19]. Indeed, the sole intervention of regular weighing (at 2–4-week intervals from 16 weeks of gestation) may reduce gestational weight gain for overweight women [20].

A range of barriers to regularly weighing pregnant women have been reported. Although many health professionals are aware of the adverse consequences of overweight/obesity in pregnancy [21] and are concerned about the impact of inappropriate GWG on patient outcomes [22–25], health professionals are reluctant to raise the issue of weight. Some staff feel that discussing weight gain causes women unnecessary anxiety during pregnancy [26], that it is ineffective [27], or that weight gain is “beyond the control of the woman” and so elect to discuss it only when the patient raises the issue [28]. However, patients find the advice they receive from clinicians helpful and that if a clinician was concerned about their weight they would also be more likely to take it seriously [29]. A number of Australian studies have demonstrated that most staff believe that they have inadequate training in the management of eGWG [28, 30, 31]. There is also some evidence that care providers are unaware of guidelines [21, 31].

To effect change in the delivery of health care it is necessary for interventions to be targeted to known barriers and designed in accordance with psychological and organisational theory that explains behavioural change. Specifically, interventions should be founded in theory that recognises the context and conditions required for behaviour and practice change to occur in health professionals. Indeed, evidenced-based behaviour change interventions have been shown to be more effective than interventions that are designed around situational or intuitive solutions [32, 33].

This study was conducted in a large Australian tertiary maternity hospital with approximately 6000 public births/year. Interviews were conducted prior to the introduction of a policy of weighing all pregnant women at every antenatal clinic visit. To investigate barriers and enablers to routine weighing of women during pregnancy at our hospital, this qualitative study explored clinicians' responses to the proposed introduction of this procedure.

## 2. Methods

All staff who would potentially provide care for women around gestational weight gain were included in this study. Obstetricians, midwives, and dietitians providing care to pregnant women at the study site were eligible to participate. Staff were informed about the study at routine meetings and via emails sent by managers inviting voluntary participation in focus group interviews. Consent was obtained immediately prior to interviews, which were recorded and transcribed by an external professional service.

An interview topic guide was developed with questions addressing four main areas: current practice; general attitudes to regular weighing; perceived patient factors that would influence weighing; perceived clinician factors that would influence weighing. Each question had a series of prompts to help elicit or expand on responses from interviewees (Table 1).

The hospital Human Research Ethics Committee reviewed and approved the study. Interviews were conducted from May to September 2015. Two authors (Tim Hasted and Shelley A. Wilkinson) independently coded the transcripts to extract key themes [34–36]. The coding was cross-checked and consensus was reached on the enablers and barriers that had been identified, as well as the current practices, general attitudes, and areas of contention amongst the different professional groups.

## 3. Results

Forty-four staff participated in four separate group interviews: 16 hospital staff midwives; 12 midwifery group practice (MGP) midwives; two dietitians; and 14 medical staff, comprising obstetric registrars and consultant obstetricians.

In addition to reporting on current weighing practices, four main themes were identified from the interviews, including (1) Systems and Resources; (2) Patient and Clinician's Personal Characteristics; (3) Advantages and Disadvantages of Routine Weighing; and (4) Evidence for Routine Weighing and Interventions.

### 3.1. Current Weighing Practice

The interviews identified wide variation in current practice. Both dietitians reported weighing all women at every visit. Some doctors reported almost never weighing women while others weighed only demonstrably obese patients. Midwives based in the hospital antenatal clinic reported varying practices, from weighing all women to rarely weighing any woman but always weighing if they were concerned about whether women were gaining appropriate weight. MGP midwives reported recording weight only at the booking in visit. When asked to assess the number of women individual clinicians had weighed in their last clinic session, dietitians had weighed all women; most midwives had weighed a minority or none. With the exception of one doctor who had weighed one woman, doctors did not record the weights of women booked into their clinics.

### 3.2. System and Resources

The following three subthemes were identified: (i) available resources; (ii) standardising and normalising the process; and (iii) documentation.

#### 3.2.1. Available Resources

Available resources concerned clinicians' access to weighing scales, the use of a hospital-developed weight tracker, and the availability of dietitians to receive referrals.

All groups interviewed identified a lack of calibrated scales as an impediment to weighing all patients at every visit:

*If they're coming in regularly and they're going to be weighed regularly, we'll have the same set. We have something that we can use for reference, and that the team and everyone else is aware of as well* (dietitian).

Hospital staff (dietitians, clinic midwives, and doctors) also commented on the lack of privacy, as the scales were in a highly visible and busy location alongside the reception counter:

*I think where the scales are now makes it hard. There's people everywhere and if there are people anxious about their weight the last thing they want to do is get on the scales in front of 15 people waiting to check in* (ANC midwife).

*Having scales in every room would be good* (ANC midwife).

All staff groups suggested that placing scales in all clinic rooms would make routine weighing easier.

The MGP midwives objected to carrying scales with them to appointments on the ground that they were too heavy/bulky and issues concerned with maintaining accurate calibration:

*If they *[pregnant women]* haven't got scales we'll just have to estimate* (MGP midwife).

All staff groups volunteered opinions about the hospital's Personalised Pregnancy Weight Tracker [37], a resource that allows women to track their own weight gain against the IOM's [12] recommendations. The consensus was that this tool allowed women to be more proactive in keeping weight gain within recommended ranges, based on self-reported BMI at the first booking visit. Doctors and midwives reported that the Tracker made it easy to discuss weight and to help educate women:

*I find it quite easy to talk about the weight tracker just because some people are quite keen to keep on track. So I'll just say, because of the - your BMI being a little bit *[unclear]*, it will trigger a referral and I can send you a weight tracker so that you can read about what foods are good in pregnancy and to also keep an eye on your weight* (MGP midwife).

However some ANC midwives felt it easier if it had already been explained:

*It's easy if the dietitians have introduced it *[weight/weighing]* first* (ANC midwife).

This was reinforced by the dietitians, who reported that some women they see had been given a weight tracker but not been told how to use it.

Midwives and doctors all expressed concerns about not usually having their first contact with women until well into the second trimester, by which time clinicians had* “already missed a great big chunk of their pregnancy” *(Doctor).

All groups discussed the need for appropriate follow-up if women were going to be weighed at every visit. The midwives and doctors stated that it was important to have sufficient numbers of dietitians to whom they could refer women:

*I guess that becomes a big point against - like a barrier to doing it is do we have somewhere to send somebody with a result* (Doctor).

*Sometimes they *[pregnant women]* bring it up themselves. I've had a couple of women go “I think I've put on a bit too much weight”. Then you talk about it and I usually send them off to a dietician* (MGP midwife).

*If it *[weight]* goes too high, that's when she can come back in and you go, now I'd like to link you in with a dietician* (MGP midwife).

#### 3.2.2. Standardising and Normalising the Process

Many staff members discussed using a single, agreed upon approach that reinforced the process as routine and did not single out specific (high) BMI women. It was suggested that staff discuss the importance of monitoring weight gain in all women and that this discussion should be documented earlier in pregnancy:

*It's strange though because it is just another measurement, just like we're measuring their blood pressure. If they've got high blood pressure, we'd react to that* (ANC midwife).

*We all run our clinics differently and that's hard for women as well […] For some people things like this aren't really brought up a lot and for other people we do talk about it a lot. There's probably other things at the other end of the spectrum that, if we could have some sort of uniformity* (ANC midwife).

While guidelines can standardise practice, dietitians stated that some maternity staff appeared to be unaware of hospital guidelines and the evidence around weight gain in pregnancy. Indeed as one doctor declared: “*Yeah we have guidelines but they really mean nothing because I'm not using them.”*

One doctor reflected on the issue of continuity of care and building rapport, stating that it was easier to weigh his private patients rather than these public patients:

*I mean in private practice it's easy, because you can see the patients dramatically changing because you've got continuity of care. It is harder in a hospital. There might be an argument for that, where they're seeing a different clinician at every visit.*

It was also thought that the way “weight” and “weighing” is discussed makes it easier to weigh women:

*They learn at their first visit at the diabetes clinic, someone would take them in and show them* [where to be weighed]* because they do their own urinalysis as well. They'd do that and they'd learn how to weigh themselves, and then, where they'd come to each week* (MGP midwife).

[At other sites it's an expectation]** –** *“it's how it's done here” *(Doctor).

Many doctors agreed that they would find it difficult to find the time to weigh women and then discuss excessive weight gain, in addition to the other routine aspects required during a consultation. Furthermore, doctors and midwifery group practice midwives suggested that women could just weigh themselves and inform clinicians at each visit:

*If they got weighed on the way in, do you know what I mean - that would be one less-so (1) it would get done 100 per cent of the time and (2) it's something that we don't have to think about doing* (Doctor).

*You have to expect that there will then be a question from them that will come to you about how's my weight going doctor? Is that okay? What would be normally expected at this gestation and there's another two minutes of your consultation time gone, about something that may not be that relevant* (Doctor).

#### 3.2.3. Documentation

The subtheme of documentation highlighted the need to have weights recorded in a systematic way and in a standard place, as well as the benefits of regular recording. This would make it easier to weigh as a reminder would exist and it would be part of the flow of each consultation. Both midwifery groups and the doctors felt that if Matrix (the computerised hospital database) had a prompt or a field that required populating (as is the case for blood pressure, fetal heart rate, and other routine measurements) this would encourage a change in practice towards routine weighing:

*Can we have one* [Matrix prompt]* for weight as well because every woman loses her weight tracker. I'm constantly going out and getting new ones and doing it from scratch and they can't remember what their previous weights were and some midwives enter them and some don't* (ANC midwife).

### 3.3. Patient and Clinician's Personal Characteristics

Participants were asked if there were characteristics that made weighing women difficult or easier.

#### 3.3.1. Patient Characteristics

Both midwifery groups agreed that it was easier to introduce the topic and to weigh women who were proactive and interested in their own weight gain or who mentioned weight themselves. The hospital staff midwives felt it was more difficult to weigh women who were “in denial,” or if their partners were with them. Doctors reported that women's anxiety was a barrier to weighing, while dietitians reported that often women with a higher BMI or those who had gained excessive amounts of weight were less likely to want to be weighed, as were those who were self-conscious or “worried about judgment.” However, according to clinicians women generally agree to be weighed with reassurance from the clinician with “*no woman ever refusing*” using this approach. One doctor suggested that women “*understand that we're mostly looking at it for a reason.*”

#### 3.3.2. Clinician Characteristics

The training that clinicians had received influenced their confidence and attitude in discussing and monitoring weight. Dietitians felt that some clinicians lacked knowledge in monitoring gestational weight gain and one doctor confirmed this:

*“I think one of the barriers for me too is not knowing how to counsel those women who are not gaining weight* [appropriately].*”*

Another doctor asked,* “What do I tell them? How do I counsel them? How do I reassure them?”*

The ANC midwives also stated that they had limited training in this area, with one midwife stating,* “So we can all take weight but what are we going to do with that information?”*

Additionally, one doctor noted they did not raise or pursue a discussion around weight due to a concern about the effectiveness of their advice.

All groups discussed staff attitudes; dietitians and MGP midwives stressed the importance of a nonjudgemental approach, although one midwife stated,* “it's very difficult to maintain that when somebody's weight is staring you in the face.”*

Additionally, both midwifery groups mentioned that their own weight influenced their approach around monitoring gestational weight gain:

[The/my]* comfort and ease to discuss is influenced by my own weight* (ANC midwife).

*I find it difficult to try and lecture somebody about healthy eating because yes, personally I, yeah - weight has always been a* [problem for me] (MGP midwife).

### 3.4. Advantages and Disadvantages of Routine Weighing

Participants pointed to a number of general advantages and disadvantages to the introduction of a policy of routine weighing.

#### 3.4.1. Advantages of Routine Weighing

Dietitians and hospital staff midwives were of the opinion that the practice would normalise weighing and reduce stigma. Staff midwives seemed to generally agree that if routine weighing was approached in the same way as measuring blood pressure, women would come to expect it. One doctor stated that it would allow for opportunistic counselling “*during the pregnancy as opposed to after it,*” although not all of the doctors agreed with the importance of this.

Some staff focussed on the benefit of having recorded weights for future research:

*There is one big advantage of weighing women that - making it compulsory each visit that they come to the antenatal clinic. I mean realistically it doesn't take that long if you've got a set of scales in the room to do a weight. It's not - and documenting it… The big advantage of that for us is that women very quickly have a very large database of women to look at* (Doctor).

*Not recording means we don't know how women are going compared with guidelines* (Doctor).

#### 3.4.2. Disadvantages of Routine Weighing

Some clinicians were concerned about the effect of the policy on the patient-practitioner relationship. Both the doctors and MGP midwives felt that women would find the practice intrusive. Doctors were concerned about women being made to feel uncomfortable at their antenatal clinic appointments:

*Women don't like getting on scales. Full stop.* (MGP midwife).

*I reckon that they'd stop turning up. They wouldn't make as many appointments. They wouldn't - yes, because they think, well you're just going to judge me* (MGP midwife).

Both dietitians and MGP midwives expressed concern that some patients might respond to closer monitoring of their weight with unhealthy eating habits. One of the dietitians was concerned that some patients might restrict their food intake and in turn “*compromise their nutritional status*”:

*I know we're trying to encourage healthy habits and healthy weight gain but I think it could actually have the opposite effect of bingeing or whatever* (MGP midwife).

### 3.5. Evidence for Routine Weighing and Interventions

Doctors were the most vocal group in questioning the evidence behind routinely weighing pregnant women and interventions for women who gained more weight than the recommended guidelines. One doctor stated,* “What's the intervention? What intervention are you going to put in place to change that?”*

Most doctors agreed that current evidence confirmed that counselling by clinicians did not result in sustained weight loss. One doctor stated,* “I'm not convinced that the evidence is strongly there that weighing all women changes outcomes.”*

Both doctors and MGP midwives agreed that while women were once routinely weighed throughout pregnancy, robust evidence to reintroduce the practice was currently lacking:

*I mean I think part of the reason it went out was really because it was having a significant negative psychological impact on women during pregnancy that was - I think that was one of the drivers certainly for it going out […] there wasn't a great deal of evidence to say that weight change at that stage was - was particularly influenced outcomes, but also there was the additional thing which was that a lot of women really found it quite intrusive* (Doctor).

*Previously we used to weigh everybody and they said, we don't need to do it anymore. So now it's like, it's being brought back in. No, we did, then we didn't […] Is there actually a need for this? If it was so important, why was it taken away in the first place?* (MGP midwife).

However, one of the dietitians disagreed, saying that* “there's a lot of evidence around gestational weight gain and the outcomes for both the mum and the bub.”*

## 4. Discussion

While many clinicians identified benefits to routine weighing, various challenges need to be addressed in order to successfully implement the process of routine weighing of all women at every antenatal visit.

### 4.1. Implementing Change and Addressing Barriers

It is apparent from these results, and as highlighted in the literature, that availability and/or dissemination of guidelines alone do not change practice [38]. Health service change theory outlines that barriers and enablers must be systematically assessed and subsequent service (behaviour) change strategies must be systematic and theory-driven [39]. French and others suggest that assessment must reflect individual, team, and organisational requirements within a robust framework (e.g., Theoretical Domains Framework or TDF) [39]. Applying this framework to our findings suggests that our barriers exist in the theoretical domains:* knowledge, skills, beliefs about capabilities, belief about consequences, and environmental context and resources.*

In formulating effective solutions to overcome these barriers (Table 2) we have employed the TDF methodology.

The training content will specifically target the barriers outlined above (knowledge of guidelines, of the evidence regarding interventions, and of available resources and referral pathways within the service) and will be informed by the implementation science literature to facilitate skill development and behaviour change. Our results suggest that staff education about the role of weighing and the evidence for available interventions would need to form part of an implementation strategy for all groups, but particularly doctors and MGP midwives.

### 4.2. Evidence for Interventions

The majority of pregnant women engage in low amounts of physical activity and they become increasingly more sedentary as their pregnancy progresses [40]. Regular physical activity may help women gain an appropriate amount of weight, reduce the risk of gestational diabetes and preeclampsia, and decrease physical complaints such as back pain [41]. While no one in this study questioned that exercise or changes to diet might control gestational weight gain, some clinicians did question the health service's ability to enable women to make these changes. While some of the participants in this study were concerned about the impact of weighing on women and on their therapeutic relationship there is no evidence that monitoring has any adverse effects for patients [42]. The evidence for routine weighing without other interventions is not conclusive. Some studies have shown a benefit [19, 20], while others have found that routine weighing alone has little or no influence on GWG [43, 44].

While strong evidence in favour of routine weighing is currently lacking, identifying eGWG may guide management and other interventions, for which there is a growing evidence base. The 2015 Cochrane review concluded that “high-quality evidence indicates that diet or exercise, or both, during pregnancy can reduce the risk of excessive GWG” [45]. The authors found that other benefits may include a lower risk of caesarean delivery, macrosomia, and neonatal respiratory morbidity, particularly for high-risk women receiving combined diet and exercise interventions. While some studies suggest that interventions encouraging healthy dietary habits or physical activity have little or no effect on GWG [46, 47], most show that educational interventions [48], behavioural interventions [49–52], counseling interventions [53], or a combination of approaches [54–56] can be successful in both obese and normal weight women.

### 4.3. Study Limitations and Areas for Future Research

This study has a number of limitations. We acknowledge that the use of a group forum for interviews did allow for some robust discussion and it is possible that more outspoken participants prevented contrary opinions and experiences to be raised by others. Nevertheless, the group format did allow for some views to be interrogated or affirmed by others and for a group consensus to form on issues after they had been debated.

We also acknowledge that we had relatively small sample sizes, especially in our dietitian group, although we interviewed 2 of the 3 dietitians working in the service.

Future research could also look at the practices and attitudes of general practitioners, who provide antenatal care for over one-quarter of Australian women and who care for nearly all of our women prior to referral to maternity services and who are therefore critical in any strategy to reduce eGWG.

The impact of the introduction of routine weighing on eGWG and subsequent fetal and maternal outcomes at our hospital will be the subject of another longitudinal study.

## 5. Conclusion

While many clinicians support the idea of routine weighing in antenatal clinic, a variety of barriers to its introduction have been identified. Clinicians raised concerns about existing resources, time constraints, and clinician and patient characteristics; clinicians' knowledge base; and access to evidence-based interventions and follow-up. Implementation strategies at our hospital will be tailored to these specific barriers to ensure all clinicians within the service are supported to be able to deliver evidence-based health care to ensure optimal outcomes for women and their babies.

## Figures and Tables

**Table 1:** Interview topic guide.

Question	Prompts
Describe the various measurements you undertake during an antenatal clinic visit.	(i) Specifically what measurements do you undertake concerning weight and calculating BMI? (ii) Do you currently weigh women? All women? When? Sometimes? (iii) Are you aware of the prompts on Matrix (electronic database) concerning weight? (iv) Is there anything that makes this easy? Is there anything that makes this difficult?

We are planning on introducing routine weighing for all pregnant women at every antenatal clinic visit. What do you think about this?	(i) Are there practical obstacles you think might make regular weighing difficult? (ii) Does weighing affect the patient-practitioner relationship? (iii) Have you had women refuse to be weighed? (iv) Does anyone have experiences at other centres that regularly weigh patients? (v) Do you think routine weighing would impact patient outcomes?

What patient factors influence whether they will be weighed in the antenatal clinic?	(i) Does the woman's baseline weight influence whether you will weigh a woman? (ii) Does a woman's interest in her own weight gain influence whether you will weigh a woman? (iii) In your last antenatal clinic session who were the women that you could or could not weigh?

What clinician factors influence whether they will be weighed in the antenatal clinic?	(i) Some studies have found that overweight clinicians find it more difficult to counsel patients about weight. Do you think a clinician's weight effects their likelihood to weigh women? (ii) Do you feel that you have enough knowledge about gestational weight gain to counsel women about their weight?

**Table 2:** Solutions.

Theoretical domains	Barriers	Solution
*Environmental context and resources*	(i) scales in public places, lack of scales in every room, MGP midwives not having access to scales (ii) lack of access to dietitians and lack of clarity about when to refer (iii) Matrix (online database) not prompting an entry for weight (iv) Time for weighing and discussing weight	(i) Funding was secured to purchase scales for every ANC room and for MGP midwives (ii) Dietitians are rostered to every antenatal clinic; referral guidelines have been updated and circulated (iii) The need for a compulsory weight prompt is being evaluated (iv) This will be evaluated after implementation and clinic appointment times altered if necessary

*Knowledge* *Skills* *Beliefs about capabilities* *Beliefs about consequences*	(i) lack of knowledge about effects of eGWG, how to counsel women on weight, and what interventions to recommend (ii) Skepticism about impact of eGWG and evidence for interventions	(i) Written materials and guidelines have been circulated and training sessions and workshops will be arranged to update staff on evidence around: the impact of eGWG, how best to approach the issue of weight with women, and interventions that have been shown to result in healthier GWG and better outcomes
